# Early speech therapy intervention in children with Autism Spectrum Disorder

**DOI:** 10.1192/j.eurpsy.2026.11159

**Published:** 2026-07-31

**Authors:** H. A. Ferreira, P. M. Pacheco, T. H. F. Santos, D. R. Molini-Avejonas

**Affiliations:** University of São Paulo, São Paulo, Brazil

## Abstract

**Introduction:**

The critical period for neurodevelopmental interventions occurs before the age of three. In this context, the DIR/Floortime model proposes a comprehensive approach to early child neurodevelopment, with an emphasis on socio-emotional development, recognized as an important predictor of well-developed social skills.

**Objectives:**

To analyze the results of a speech-language intervention based on the principles of the DIR/Floortime model in very early childhood in children diagnosed with Autism Spectrum Disorder (ASD).

**Methods:**

This is a longitudinal, quantitative, and prospective study with both direct and indirect interventions. The target population consisted of children between one and three years of age with atypical language development associated with ASD. The initial assessment included a Clinical History, the Pragmatics protocol of the ABFW Child Language Test (2004), and the Functional Emotional Assessment Scale (FEAS). Subsequently, 24 weekly speech-language therapy sessions were conducted based on the DIR/Floortime model principles, combined with biweekly parental guidance sessions. At the end of the process, two final assessment sessions were carried out using the same instruments as in the initial phase, allowing comparison of pre- and post-intervention data. This research project was approved by the Ethics Committee of the Clinical Hospital of the University of São Paulo Medical School, approval number 3.633.171 / CAEE 15171519.2.0000.0065.

**Results:**

A total of 20 children completed the study, with a mean age of 36 months at the final assessment, with a higher prevalence (80%) of males. Among those who completed the study, 90% already had a diagnosis of Childhood Autism, while the others were undergoing diagnostic investigation. In the Pragmatics test, an average increase of 0.8 communicative acts per minute was observed, as well as a 6.66% increase in communicative space occupation — both statistically significant (p-value < 0.05). Regarding the Functional Emotional Assessment Scale (FEAS), an average improvement of 1.25 points was observed across all evaluated subsystems, also with statistical significance (p-value < 0.05). The subsystems showing the greatest change were: “Self-regulation and interest in the world,” “Forming relationships, bonds, and engagement,”and “Two-way intentional communication”. Furthermore, a moderate positive correlation was found between the “two-way intentional communication” aspect and the number of communicative acts expressed per minute — indicating that the greater the intentional communication ability, the higher the frequency of communicative acts.

**Conclusions:**

The analysis of pre- and post-intervention results revealed consistent and statistically significant progress in the communicative abilities of the participating children.

**Disclosure of Interest:**

None Declared

